# *N*-[(2,6-Di­ethyl­phen­yl)carbamo­thio­yl]-2,2-di­phenyl­acetamide

**DOI:** 10.1107/S1600536813013354

**Published:** 2013-05-25

**Authors:** Mohd Sukeri Mohd Yusof, Nur Rafikah Razali, Suhana Arshad, Azhar Abdul Rahman, Ibrahim Abdul Razak

**Affiliations:** a Department of Chemical Sciences, Faculty of Science and Technology, Universiti Malaysia Terengganu, Mengabang Telipot, 21030 Kuala Terengganu, Malaysia; b School of Physics, Universiti Sains Malaysia, 11800 USM, Penang, Malaysia

## Abstract

In the title compound, C_25_H_26_N_2_OS, the diethyl-substituted benzene ring forms dihedral angles of 67.38 (9) and 55.32 (9)° with the terminal benzene rings. The mol­ecule adopts a *trans*–*cis* conformation with respect to the orientations of the di­phenyl­methane and 1,3-di­ethyl­benzene groups with respect to the S atom across the C—N bonds. This conformation is stabilized by an intra­molecular N—H⋯O hydrogen bond, which generates an *S*(6) ring. In the crystal, pairs of N—H⋯S hydrogen bonds link the mol­ecules into inversion dimers, forming *R*_2_^2^(6) loops. The dimer linkage is reinforced by a pair of C—H⋯S hydrogen bonds, which generate *R*_2_^2^(8) loops. Weak C—H⋯π and π–π [centroid–centroid seperation = 3.8821 (10) Å] inter­actions also occur in the crystal structure.

## Related literature

For related structures and backgroud to thio­urea derivatives, see: Yusof *et al.* (2012*a* ▶,*b* ▶). For hydrogen-bond motifs, see: Bernstein *et al.* (1995 ▶). For the stability of the temperature controller used for the data collection, see: Cosier & Glazer (1986 ▶).
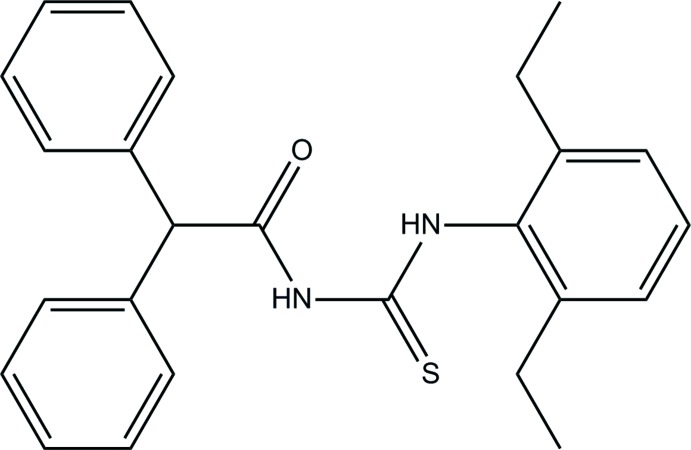


## Experimental

### 

#### Crystal data

C_25_H_26_N_2_OS
*M* *_r_* = 402.54
Triclinic, 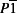

*a* = 8.0091 (1) Å
*b* = 11.7289 (2) Å
*c* = 11.8923 (2) Å
α = 79.008 (1)°
β = 80.628 (1)°
γ = 83.936 (1)°
*V* = 1078.79 (3) Å^3^
*Z* = 2
Mo *K*α radiation
μ = 0.17 mm^−1^
*T* = 100 K
0.41 × 0.17 × 0.08 mm

#### Data collection

Bruker SMART APEXII CCD diffractometer
Absorption correction: multi-scan (*SADABS*; Bruker, 2009 ▶) *T*_min_ = 0.934, *T*_max_ = 0.987
20294 measured reflections
3767 independent reflections
3123 reflections with *I* > 2σ(*I*)
*R*_int_ = 0.037

#### Refinement

*R*[*F* ^2^ > 2σ(*F* ^2^)] = 0.038
*wR*(*F* ^2^) = 0.087
*S* = 1.06
3767 reflections
272 parameters
H atoms treated by a mixture of independent and constrained refinement
Δρ_max_ = 0.27 e Å^−3^
Δρ_min_ = −0.25 e Å^−3^

### 

Data collection: *APEX2* (Bruker, 2009 ▶); cell refinement: *SAINT* (Bruker, 2009 ▶); data reduction: *SAINT*; program(s) used to solve structure: *SHELXTL* (Sheldrick, 2008 ▶); program(s) used to refine structure: *SHELXTL*; molecular graphics: *SHELXTL*; software used to prepare material for publication: *SHELXTL* and *PLATON* (Spek, 2009 ▶).

## Supplementary Material

Crystal structure: contains datablock(s) global, I. DOI: 10.1107/S1600536813013354/hb7080sup1.cif

Structure factors: contains datablock(s) I. DOI: 10.1107/S1600536813013354/hb7080Isup2.hkl

Supplementary material file. DOI: 10.1107/S1600536813013354/hb7080Isup3.cml

Additional supplementary materials:  crystallographic information; 3D view; checkCIF report

## Figures and Tables

**Table 1:** Hydrogen-bond geometry (Å, °) *Cg*1 is the centroid of the C1–C6 benzene ring.

*D*—H⋯*A*	*D*—H	H⋯*A*	*D*⋯*A*	*D*—H⋯*A*
N2—H1*N*2⋯O1	0.86 (2)	1.96 (2)	2.6702 (19)	140 (2)
N1—H1*N*1⋯S1^i^	0.85 (2)	2.59 (2)	3.4225 (16)	167.4 (18)
C7—H7*A*⋯S1^i^	1.00	2.64	3.6172 (17)	165
C10—H10*A*⋯*Cg*1^ii^	0.95	2.56	3.3859 (19)	146

Symmetry codes: (i) 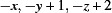; (ii) 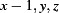.

**Fig. 1.**
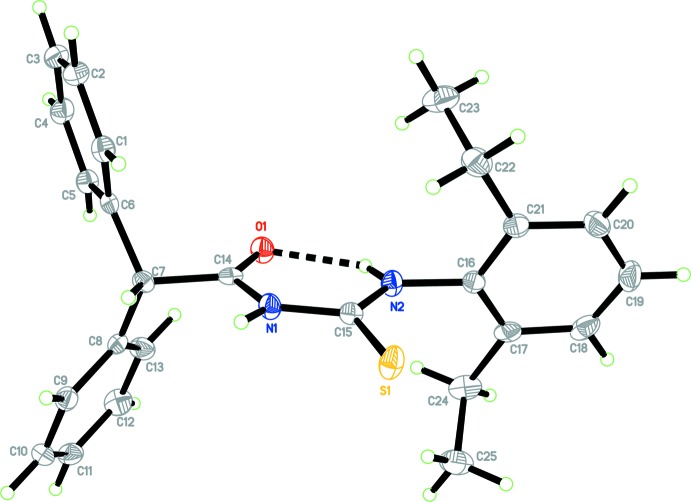
The molecular structure of the title compound with 50% probability displacement ellipsoids.

**Fig. 2.**
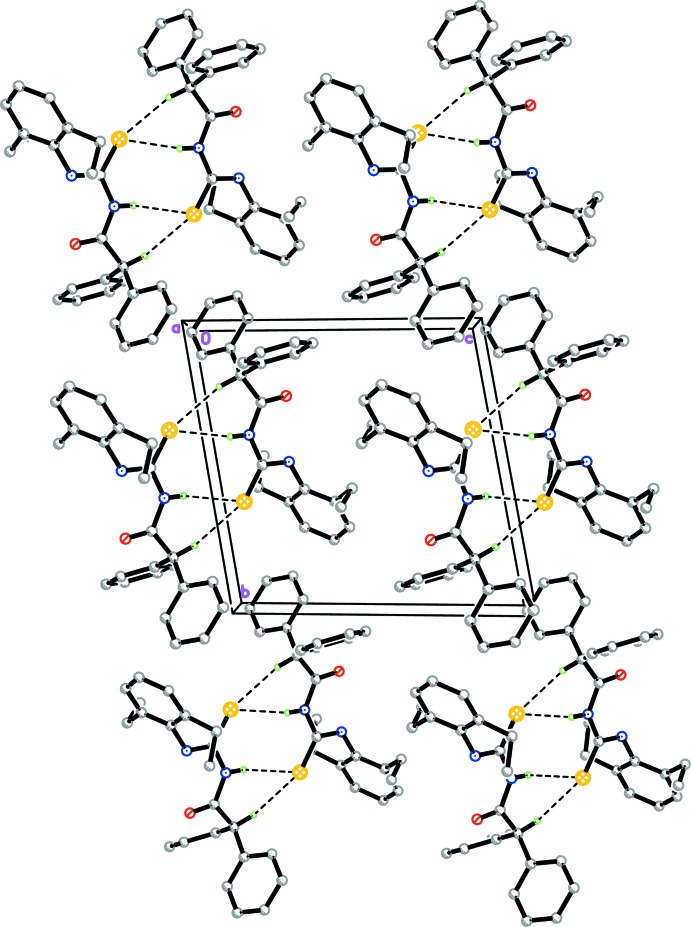
The crystal packing of the title compound. The H atoms not involved in the intermolecular interactions (dashed lines) have been omitted for clarity.

## supplementary crystallographic information

### Comment

As part of our ongoing studies of thiourea derivatives (Yusof *et al.*, 2012*a*,*b*), we now describe the structure of the title compound, (I), (Fig. 1).

The bond lengths and angles are comparable to those in related structure (Yusof *et al.*, 2012*a*,*b*). The diethyl-substituted benzene ring (C16–C21) forms dihedral angles of 67.38 (9) and 55.32 (9)° with the terminal benzene rings (C1–C6 & C8–C13), respectively. The molecule adopts a *trans*-*cis* conformation with respect to the position of diphenylmethane and 1,3-diethylbenzene groups to the sulfur (S1) atom across the C–N bonds, respectively. These configuration further resulting in an *S*(6) graph-set motif (Bernstein *et al.*, 1995) *via* intra-molecular N2—H1N2···O1 hydrogen bond (Table 1).

In the crystal (Fig. 2), molecules are linked into dimers *via* N1—H1N1···S1 and C7—H7A···S1 hydrogen bonds (Table 1), generating *R*^2^_2_(6) and *R*^2^_2_(8) loops. C10—H10A···*Cg*1 (Table 1) interactions and π–π interactions of *Cg*1···*Cg*1 = 3.8821 (10) Å (symmetry code: -*x*, 2-*y*, 2-*z*) further stabilized the crystal structure (*Cg*1 is the centroid of C1–C6).

### Experimental

An acetone (30 ml) solution of 2,6-diethylaniline (2.01 g, 13.5 mmol) was added to a round-bottom flask containing 2,2-diphenylacetyl chloride (3.10 g, 13.5 mmol) and ammonium thiocyanate (1.03 g, 13.5 mmol). The mixture was put at reflux for 2.5 h then filtered off and left to evaporate at room temperature. The colourless precipitate obtained was washed with water and cold ethanol. Colourless plates were obtained by recrystallization of the precipitate from MeOH solution.

### Refinement

N-bound H atoms was located from the difference map and refined freely, [N–H = 0.85 (2) and 0.86 (2) Å]. The remaining H atoms were positioned geometrically [C–H = 0.95–1.00 Å] and refined using a riding model with *U*_iso_(H) = 1.2 or 1.5 *U*_eq_(C).A rotating group model was applied to the methyl groups. In the final refinement two outliers were omitted (6 - 2 8 and 5 - 3 6).

### Crystal data

**Table:**

C_25_H_26_N_2_OS	*Z* = 2
*M**_r_* = 402.54	*F*(000) = 428
Triclinic, *P*1	*D*_x_ = 1.239 Mg m^−^^3^
Hall symbol: -P 1	Mo *K*α radiation, λ = 0.71073 Å
*a* = 8.0091 (1) Å	Cell parameters from 7540 reflections
*b* = 11.7289 (2) Å	θ = 2.7–31.3°
*c* = 11.8923 (2) Å	µ = 0.17 mm^−^^1^
α = 79.008 (1)°	*T* = 100 K
β = 80.628 (1)°	Plate, colourless
γ = 83.936 (1)°	0.41 × 0.17 × 0.08 mm
*V* = 1078.79 (3) Å^3^	

### Data collection

**Table:**

Bruker SMART APEXII CCD diffractometer	3767 independent reflections
Radiation source: fine-focus sealed tube	3123 reflections with *I* > 2σ(*I*)
Graphite monochromator	*R*_int_ = 0.037
φ and ω scans	θ_max_ = 25.0°, θ_min_ = 1.8°
Absorption correction: multi-scan (*SADABS*; Bruker, 2009)	*h* = −9→9
*T*_min_ = 0.934, *T*_max_ = 0.987	*k* = −13→13
20294 measured reflections	*l* = −13→14

### Refinement

**Table:**

Refinement on *F*^2^	Primary atom site location: structure-invariant direct methods
Least-squares matrix: full	Secondary atom site location: difference Fourier map
*R*[*F*^2^ > 2σ(*F*^2^)] = 0.038	Hydrogen site location: inferred from neighbouring sites
*wR*(*F*^2^) = 0.087	H atoms treated by a mixture of independent and constrained refinement
*S* = 1.06	*w* = 1/[σ^2^(*F*_o_^2^) + (0.0256*P*)^2^ + 0.7158*P*] where *P* = (*F*_o_^2^ + 2*F*_c_^2^)/3
3767 reflections	(Δ/σ)_max_ = 0.001
272 parameters	Δρ_max_ = 0.27 e Å^−^^3^
0 restraints	Δρ_min_ = −0.25 e Å^−^^3^

### Special details

**Table:**

Experimental. The crystal was placed in the cold stream of an Oxford Cryosystems Cobra open-flow nitrogen cryostat (Cosier & Glazer, 1986) operating at 100.0 (1) K.
Geometry. All e.s.d.'s (except the e.s.d. in the dihedral angle between two l.s. planes) are estimated using the full covariance matrix. The cell e.s.d.'s are taken into account individually in the estimation of e.s.d.'s in distances, angles and torsion angles; correlations between e.s.d.'s in cell parameters are only used when they are defined by crystal symmetry. An approximate (isotropic) treatment of cell e.s.d.'s is used for estimating e.s.d.'s involving l.s. planes.
Refinement. Refinement of *F*^2^ against ALL reflections. The weighted *R*-factor *wR* and goodness of fit *S* are based on *F*^2^, conventional *R*-factors *R* are based on *F*, with *F* set to zero for negative *F*^2^. The threshold expression of *F*^2^ > σ(*F*^2^) is used only for calculating *R*-factors(gt) *etc*. and is not relevant to the choice of reflections for refinement. *R*-factors based on *F*^2^ are statistically about twice as large as those based on *F*, and *R*- factors based on ALL data will be even larger.

### Fractional atomic coordinates and isotropic or equivalent isotropic displacement parameters (Å^2^)

**Table:**

	*x*	*y*	*z*	*U*_iso_*/*U*_eq_	
S1	0.14725 (6)	0.37823 (4)	0.90032 (4)	0.02277 (14)	
O1	0.23366 (16)	0.74355 (10)	0.69663 (10)	0.0209 (3)	
N1	0.1116 (2)	0.60788 (12)	0.84279 (13)	0.0175 (3)	
H1N1	0.041 (3)	0.6010 (17)	0.9051 (18)	0.027 (6)*	
N2	0.32515 (19)	0.51541 (13)	0.72816 (13)	0.0176 (3)	
H1N2	0.334 (3)	0.5850 (19)	0.6903 (18)	0.030 (6)*	
C1	0.1681 (2)	0.88822 (15)	0.95814 (15)	0.0192 (4)	
H1A	0.1494	0.8182	1.0122	0.023*	
C2	0.2650 (2)	0.96979 (16)	0.98238 (16)	0.0214 (4)	
H2A	0.3124	0.9555	1.0525	0.026*	
C3	0.2921 (2)	1.07207 (16)	0.90366 (16)	0.0226 (4)	
H3A	0.3599	1.1275	0.9190	0.027*	
C4	0.2204 (2)	1.09329 (15)	0.80297 (16)	0.0217 (4)	
H4A	0.2377	1.1640	0.7498	0.026*	
C5	0.1228 (2)	1.01165 (15)	0.77866 (16)	0.0191 (4)	
H5A	0.0733	1.0272	0.7094	0.023*	
C6	0.0981 (2)	0.90785 (14)	0.85548 (15)	0.0151 (4)	
C7	0.0022 (2)	0.81080 (14)	0.83243 (14)	0.0154 (4)	
H7A	−0.0538	0.7715	0.9094	0.018*	
C8	−0.1370 (2)	0.84747 (14)	0.75624 (15)	0.0165 (4)	
C9	−0.3053 (2)	0.84527 (15)	0.80968 (16)	0.0194 (4)	
H9A	−0.3293	0.8223	0.8913	0.023*	
C10	−0.4384 (2)	0.87616 (15)	0.74551 (16)	0.0218 (4)	
H10A	−0.5525	0.8745	0.7832	0.026*	
C11	−0.4046 (3)	0.90924 (16)	0.62722 (17)	0.0239 (4)	
H11A	−0.4954	0.9304	0.5831	0.029*	
C12	−0.2384 (3)	0.91175 (17)	0.57251 (16)	0.0261 (5)	
H12A	−0.2155	0.9349	0.4908	0.031*	
C13	−0.1047 (2)	0.88063 (16)	0.63647 (16)	0.0219 (4)	
H13A	0.0091	0.8820	0.5982	0.026*	
C14	0.1283 (2)	0.71976 (15)	0.78288 (15)	0.0159 (4)	
C15	0.2009 (2)	0.50521 (15)	0.81792 (15)	0.0170 (4)	
C16	0.4354 (2)	0.41906 (14)	0.69377 (15)	0.0164 (4)	
C17	0.4107 (2)	0.37685 (15)	0.59603 (15)	0.0196 (4)	
C18	0.5229 (3)	0.28617 (16)	0.56247 (17)	0.0271 (5)	
H18A	0.5095	0.2562	0.4959	0.032*	
C19	0.6536 (3)	0.23894 (17)	0.62419 (19)	0.0310 (5)	
H19A	0.7280	0.1764	0.6006	0.037*	
C20	0.6758 (2)	0.28280 (16)	0.72028 (18)	0.0258 (5)	
H20A	0.7659	0.2500	0.7622	0.031*	
C21	0.5685 (2)	0.37423 (15)	0.75657 (15)	0.0195 (4)	
C22	0.6019 (3)	0.42551 (17)	0.85727 (16)	0.0260 (5)	
H22A	0.6723	0.3677	0.9053	0.031*	
H22B	0.4926	0.4417	0.9060	0.031*	
C23	0.6922 (3)	0.53785 (18)	0.81866 (18)	0.0333 (5)	
H23A	0.7139	0.5662	0.8869	0.050*	
H23B	0.6204	0.5969	0.7745	0.050*	
H23C	0.8001	0.5226	0.7698	0.050*	
C24	0.2659 (3)	0.42388 (17)	0.52918 (17)	0.0278 (5)	
H24A	0.3007	0.4157	0.4471	0.033*	
H24B	0.2414	0.5079	0.5318	0.033*	
C25	0.1047 (3)	0.36140 (18)	0.57675 (19)	0.0326 (5)	
H25A	0.0156	0.3941	0.5296	0.049*	
H25B	0.0670	0.3719	0.6570	0.049*	
H25C	0.1280	0.2781	0.5741	0.049*	

### Atomic displacement parameters (Å^2^)

**Table:**

	*U*^11^	*U*^22^	*U*^33^	*U*^12^	*U*^13^	*U*^23^
S1	0.0282 (3)	0.0146 (2)	0.0217 (3)	−0.0026 (2)	0.0066 (2)	−0.00198 (18)
O1	0.0191 (7)	0.0186 (6)	0.0223 (7)	−0.0033 (5)	0.0043 (6)	−0.0022 (5)
N1	0.0161 (8)	0.0163 (8)	0.0178 (8)	−0.0019 (6)	0.0040 (7)	−0.0024 (6)
N2	0.0186 (9)	0.0137 (8)	0.0181 (8)	−0.0010 (6)	0.0015 (7)	−0.0009 (6)
C1	0.0192 (10)	0.0180 (9)	0.0191 (10)	0.0007 (8)	−0.0016 (8)	−0.0023 (7)
C2	0.0208 (11)	0.0249 (10)	0.0210 (10)	−0.0003 (8)	−0.0064 (8)	−0.0079 (8)
C3	0.0195 (11)	0.0236 (10)	0.0270 (11)	−0.0042 (8)	−0.0011 (8)	−0.0106 (8)
C4	0.0233 (11)	0.0166 (9)	0.0241 (10)	−0.0039 (8)	0.0012 (8)	−0.0032 (8)
C5	0.0187 (10)	0.0194 (9)	0.0188 (9)	−0.0005 (8)	−0.0013 (8)	−0.0039 (7)
C6	0.0110 (9)	0.0165 (9)	0.0178 (9)	0.0006 (7)	0.0004 (7)	−0.0057 (7)
C7	0.0133 (9)	0.0166 (9)	0.0152 (9)	−0.0021 (7)	0.0001 (7)	−0.0012 (7)
C8	0.0162 (10)	0.0125 (8)	0.0218 (10)	−0.0026 (7)	−0.0015 (8)	−0.0058 (7)
C9	0.0203 (10)	0.0183 (9)	0.0198 (10)	−0.0032 (8)	0.0009 (8)	−0.0059 (7)
C10	0.0140 (10)	0.0232 (10)	0.0297 (11)	−0.0012 (8)	−0.0026 (8)	−0.0092 (8)
C11	0.0220 (11)	0.0230 (10)	0.0304 (11)	−0.0007 (8)	−0.0115 (9)	−0.0083 (8)
C12	0.0307 (12)	0.0293 (11)	0.0188 (10)	−0.0037 (9)	−0.0059 (9)	−0.0026 (8)
C13	0.0186 (11)	0.0255 (10)	0.0214 (10)	−0.0052 (8)	−0.0003 (8)	−0.0038 (8)
C14	0.0143 (10)	0.0173 (9)	0.0180 (9)	−0.0050 (7)	−0.0052 (8)	−0.0035 (7)
C15	0.0155 (10)	0.0180 (9)	0.0182 (9)	−0.0018 (7)	−0.0031 (8)	−0.0043 (7)
C16	0.0144 (10)	0.0157 (9)	0.0180 (9)	−0.0033 (7)	0.0016 (7)	−0.0026 (7)
C17	0.0173 (10)	0.0222 (10)	0.0191 (10)	−0.0107 (8)	0.0030 (8)	−0.0027 (8)
C18	0.0269 (12)	0.0276 (11)	0.0285 (11)	−0.0135 (9)	0.0090 (9)	−0.0142 (9)
C19	0.0224 (12)	0.0218 (10)	0.0470 (13)	−0.0013 (9)	0.0087 (10)	−0.0136 (9)
C20	0.0164 (10)	0.0225 (10)	0.0372 (12)	−0.0012 (8)	−0.0027 (9)	−0.0030 (9)
C21	0.0185 (10)	0.0176 (9)	0.0218 (10)	−0.0050 (8)	−0.0010 (8)	−0.0018 (8)
C22	0.0252 (11)	0.0288 (11)	0.0241 (10)	0.0000 (9)	−0.0078 (9)	−0.0027 (8)
C23	0.0348 (13)	0.0402 (12)	0.0304 (12)	−0.0117 (10)	−0.0077 (10)	−0.0125 (10)
C24	0.0294 (12)	0.0324 (11)	0.0229 (10)	−0.0111 (9)	−0.0050 (9)	−0.0022 (9)
C25	0.0254 (12)	0.0320 (11)	0.0409 (13)	−0.0077 (9)	−0.0107 (10)	0.0003 (10)

### Geometric parameters (Å, º)

**Table:**

S1—C15	1.6748 (17)	C11—C12	1.384 (3)
O1—C14	1.225 (2)	C11—H11A	0.9500
N1—C14	1.377 (2)	C12—C13	1.391 (3)
N1—C15	1.391 (2)	C12—H12A	0.9500
N1—H1N1	0.85 (2)	C13—H13A	0.9500
N2—C15	1.332 (2)	C16—C17	1.396 (3)
N2—C16	1.439 (2)	C16—C21	1.401 (3)
N2—H1N2	0.86 (2)	C17—C18	1.393 (3)
C1—C2	1.391 (3)	C17—C24	1.510 (3)
C1—C6	1.395 (2)	C18—C19	1.383 (3)
C1—H1A	0.9500	C18—H18A	0.9500
C2—C3	1.387 (3)	C19—C20	1.383 (3)
C2—H2A	0.9500	C19—H19A	0.9500
C3—C4	1.380 (3)	C20—C21	1.391 (3)
C3—H3A	0.9500	C20—H20A	0.9500
C4—C5	1.396 (3)	C21—C22	1.510 (3)
C4—H4A	0.9500	C22—C23	1.527 (3)
C5—C6	1.387 (2)	C22—H22A	0.9900
C5—H5A	0.9500	C22—H22B	0.9900
C6—C7	1.527 (2)	C23—H23A	0.9800
C7—C14	1.525 (2)	C23—H23B	0.9800
C7—C8	1.525 (2)	C23—H23C	0.9800
C7—H7A	1.0000	C24—C25	1.526 (3)
C8—C13	1.392 (2)	C24—H24A	0.9900
C8—C9	1.394 (2)	C24—H24B	0.9900
C9—C10	1.389 (3)	C25—H25A	0.9800
C9—H9A	0.9500	C25—H25B	0.9800
C10—C11	1.376 (3)	C25—H25C	0.9800
C10—H10A	0.9500		
			
C14—N1—C15	128.46 (15)	O1—C14—N1	122.87 (16)
C14—N1—H1N1	115.8 (14)	O1—C14—C7	123.13 (15)
C15—N1—H1N1	115.7 (14)	N1—C14—C7	113.98 (14)
C15—N2—C16	124.03 (15)	N2—C15—N1	116.69 (15)
C15—N2—H1N2	114.5 (14)	N2—C15—S1	124.24 (14)
C16—N2—H1N2	121.5 (14)	N1—C15—S1	119.07 (13)
C2—C1—C6	120.85 (16)	C17—C16—C21	122.22 (17)
C2—C1—H1A	119.6	C17—C16—N2	118.95 (16)
C6—C1—H1A	119.6	C21—C16—N2	118.77 (16)
C3—C2—C1	119.62 (17)	C18—C17—C16	117.69 (18)
C3—C2—H2A	120.2	C18—C17—C24	119.97 (17)
C1—C2—H2A	120.2	C16—C17—C24	122.32 (17)
C4—C3—C2	119.93 (17)	C19—C18—C17	121.23 (18)
C4—C3—H3A	120.0	C19—C18—H18A	119.4
C2—C3—H3A	120.0	C17—C18—H18A	119.4
C3—C4—C5	120.51 (17)	C20—C19—C18	119.91 (18)
C3—C4—H4A	119.7	C20—C19—H19A	120.0
C5—C4—H4A	119.7	C18—C19—H19A	120.0
C6—C5—C4	120.08 (17)	C19—C20—C21	121.10 (19)
C6—C5—H5A	120.0	C19—C20—H20A	119.4
C4—C5—H5A	120.0	C21—C20—H20A	119.4
C5—C6—C1	118.98 (16)	C20—C21—C16	117.83 (17)
C5—C6—C7	123.53 (16)	C20—C21—C22	120.08 (18)
C1—C6—C7	117.46 (15)	C16—C21—C22	122.03 (16)
C14—C7—C8	109.72 (14)	C21—C22—C23	112.66 (16)
C14—C7—C6	109.54 (14)	C21—C22—H22A	109.1
C8—C7—C6	116.82 (14)	C23—C22—H22A	109.1
C14—C7—H7A	106.7	C21—C22—H22B	109.1
C8—C7—H7A	106.7	C23—C22—H22B	109.1
C6—C7—H7A	106.7	H22A—C22—H22B	107.8
C13—C8—C9	118.51 (17)	C22—C23—H23A	109.5
C13—C8—C7	123.51 (16)	C22—C23—H23B	109.5
C9—C8—C7	117.97 (15)	H23A—C23—H23B	109.5
C10—C9—C8	121.07 (17)	C22—C23—H23C	109.5
C10—C9—H9A	119.5	H23A—C23—H23C	109.5
C8—C9—H9A	119.5	H23B—C23—H23C	109.5
C11—C10—C9	119.79 (17)	C17—C24—C25	112.69 (16)
C11—C10—H10A	120.1	C17—C24—H24A	109.1
C9—C10—H10A	120.1	C25—C24—H24A	109.1
C10—C11—C12	120.03 (18)	C17—C24—H24B	109.1
C10—C11—H11A	120.0	C25—C24—H24B	109.1
C12—C11—H11A	120.0	H24A—C24—H24B	107.8
C11—C12—C13	120.37 (18)	C24—C25—H25A	109.5
C11—C12—H12A	119.8	C24—C25—H25B	109.5
C13—C12—H12A	119.8	H25A—C25—H25B	109.5
C12—C13—C8	120.23 (17)	C24—C25—H25C	109.5
C12—C13—H13A	119.9	H25A—C25—H25C	109.5
C8—C13—H13A	119.9	H25B—C25—H25C	109.5
			
C6—C1—C2—C3	0.1 (3)	C6—C7—C14—O1	−54.8 (2)
C1—C2—C3—C4	1.2 (3)	C8—C7—C14—N1	−104.38 (17)
C2—C3—C4—C5	−1.0 (3)	C6—C7—C14—N1	126.15 (16)
C3—C4—C5—C6	−0.4 (3)	C16—N2—C15—N1	176.91 (16)
C4—C5—C6—C1	1.7 (3)	C16—N2—C15—S1	−3.3 (3)
C4—C5—C6—C7	−176.24 (16)	C14—N1—C15—N2	3.8 (3)
C2—C1—C6—C5	−1.6 (3)	C14—N1—C15—S1	−176.01 (15)
C2—C1—C6—C7	176.50 (16)	C15—N2—C16—C17	104.9 (2)
C5—C6—C7—C14	96.32 (19)	C15—N2—C16—C21	−77.9 (2)
C1—C6—C7—C14	−81.68 (18)	C21—C16—C17—C18	0.7 (3)
C5—C6—C7—C8	−29.2 (2)	N2—C16—C17—C18	177.83 (15)
C1—C6—C7—C8	152.83 (16)	C21—C16—C17—C24	179.14 (16)
C14—C7—C8—C13	−49.6 (2)	N2—C16—C17—C24	−3.8 (2)
C6—C7—C8—C13	75.8 (2)	C16—C17—C18—C19	0.4 (3)
C14—C7—C8—C9	129.11 (16)	C24—C17—C18—C19	−177.99 (17)
C6—C7—C8—C9	−105.49 (18)	C17—C18—C19—C20	−0.8 (3)
C13—C8—C9—C10	−0.5 (3)	C18—C19—C20—C21	0.0 (3)
C7—C8—C9—C10	−179.22 (15)	C19—C20—C21—C16	1.1 (3)
C8—C9—C10—C11	0.2 (3)	C19—C20—C21—C22	−176.21 (17)
C9—C10—C11—C12	0.0 (3)	C17—C16—C21—C20	−1.5 (3)
C10—C11—C12—C13	0.2 (3)	N2—C16—C21—C20	−178.60 (15)
C11—C12—C13—C8	−0.5 (3)	C17—C16—C21—C22	175.75 (16)
C9—C8—C13—C12	0.6 (3)	N2—C16—C21—C22	−1.3 (2)
C7—C8—C13—C12	179.27 (16)	C20—C21—C22—C23	99.3 (2)
C15—N1—C14—O1	−2.0 (3)	C16—C21—C22—C23	−77.9 (2)
C15—N1—C14—C7	177.14 (16)	C18—C17—C24—C25	90.5 (2)
C8—C7—C14—O1	74.7 (2)	C16—C17—C24—C25	−87.9 (2)

### Hydrogen-bond geometry (Å, º)

Cg1 is the centroid of the C1–C6 benzene ring.

**Table:**

*D*—H···*A*	*D*—H	H···*A*	*D*···*A*	*D*—H···*A*
N2—H1*N*2···O1	0.86 (2)	1.96 (2)	2.6702 (19)	140 (2)
N1—H1*N*1···S1^i^	0.85 (2)	2.59 (2)	3.4225 (16)	167.4 (18)
C7—H7*A*···S1^i^	1.00	2.64	3.6172 (17)	165
C10—H10*A*···*Cg*1^ii^	0.95	2.56	3.3859 (19)	146

Symmetry codes: (i) −*x*, −*y*+1, −*z*+2; (ii) *x*−1, *y*, *z*.

## References

[bb1] Bernstein, J., Davis, R. E., Shimoni, L. & Chang, N.-L. (1995). *Angew. Chem. Int. Ed. Engl.* **34**, 1555–1573.

[bb2] Bruker (2009). *SADABS*, *APEX2* and *SAINT* Bruker AXS Inc., Madison, Wisconsin, USA.

[bb3] Cosier, J. & Glazer, A. M. (1986). *J. Appl. Cryst.* **19**, 105–107.

[bb4] Sheldrick, G. M. (2008). *Acta Cryst.* A**64**, 112–122. 10.1107/S0108767307043930 18156677

[bb5] Spek, A. L. (2009). *Acta Cryst.* D**65**, 148–155. 10.1107/S090744490804362X PMC2631630 19171970

[bb6] Yusof, M. S. M., Arshad, S., Razak, I. A. & Rahman, A. A. (2012*b*). *Acta Cryst.* E**68**, o2670. 10.1107/S1600536812034174 PMC3435693 22969564

[bb7] Yusof, M. S. M., Mutalib, S. F. A., Arshad, S. & Razak, I. A. (2012*a*). *Acta Cryst.* E**68**, o982. 10.1107/S1600536812009233 PMC3343953 22590034

